# Tumor Inhibition by DepoVax-Based Cancer Vaccine Is Accompanied by Reduced Regulatory/Suppressor Cell Proliferation and Tumor Infiltration

**DOI:** 10.1155/2013/753427

**Published:** 2013-03-07

**Authors:** Mohan Karkada, Tara Quinton, Rachelle Blackman, Marc Mansour

**Affiliations:** ^1^Immunovaccine Inc., Division of Immunology, 1344 Summer Street, Halifax, NS, Canada B3H 0A8; ^2^Department of Microbiology and Immunology, Dalhousie University, 5850 College Street, Halifax, NS, Canada B3H 4R2

## Abstract

A successful cancer vaccine needs to overcome the effects of immune-suppressor cells such as Treg lymphocytes, suppressive cytokine-secreting Tr1 cells, and myeloid-derived suppressor cells (MDSCs), while enhancing tumor-specific immune responses. Given the relative poor efficacy associated with current cancer vaccines, a novel vaccine platform called DepoVax^TM^ (DPX) was developed. C3 tumor-challenged mice were immunized with HPV-E7 peptide in DPX- or conventional-emulsion- (CE-) based vaccine. While control mice showed marked increase in Treg/MDSCs in spleen and blood, in mice treated with DPX-E7 the levels remained similar to tumor-free naive mice. Such differences were also seen within the tumor. Antigen-specific IL10-secreting CD4/CD8 T cells and TGF-**β** ^+^CD8^+^ T cell frequencies were increased significantly in CE-treated and control mice in contrast to DPX-E7-immunized mice. Analysis of tumor-infiltrating cells revealed higher frequency of suppressor cells in untreated controls than in DPX-E7 group while the converse was true for tumor-infiltrating CD8 T cells. Immunization of tumor-bearing HLA-A2 transgenic mice with human vaccine DPX-0907, a peptide-based vaccine for breast/ovarian/prostate cancers, showed efficient induction of immune response to cancer peptides despite the presence of suppressor cells. Thus, this study provides the rationale for using DPX-based cancer vaccines in immune-suppressed cancer patients, to induce effective anticancer immunity.

## 1. Introduction

Lack of effective cancer treatments and the emergence of drug resistance in progressively growing tumors make cancer immunotherapy a viable alternative to treat and manage metastatic diseases [1]. Synthetic peptide-based vaccines that are capable of inducing specific T cell-mediated immunity are emerging as attractive therapeutic strategies for cancers. While such vaccines have proven rather effective in diverse animal studies, multiple human clinical trials implementing this approach have thus far yielded limited success [2–5]. Although many of these trials were able to elicit significant number of tumor antigen-specific T cells in cancer patients, clinical regressions of disease remain rare [6]. Although incompletely understood, the lack of clinical efficacy for peptide-based vaccines may be related to several factors such as poor immunogenicity of the chosen peptides, insufficient numbers or appropriate functional polarity of responder T cells, inefficient trafficking of effector cells into tumor microenvironment, and, more importantly, tumor-induced increase in the number of immunosuppressive regulatory cells. Thus, there remains considerable room for improvement in cancer vaccine design in order to maximize the chances of clinical benefit to the patients.

Many studies have identified highly diverse subset of tumor infiltrating leukocytes, both with pro- and antitumor functions, among lymphocyte, NK cells, macrophages, and neutrophils [7]. Naturally occurring and inducible CD4^+^CD25^+^Foxp3^+^ T regulatory lymphocytes (Treg), suppressive cytokine-secreting Tr1 cells, protect the host from autoimmune disease by suppressing self-reactive cells. However, these cells can also block anti-tumor/antimicrobial immune responses. Particularly in the context of cancer, Treg/Tr1-cell frequencies and function are important because increased numbers might dampen anti-cancer immune responses and favor tumor growth [8–10]. In addition to Treg and Tr1 cells, myeloid derived suppressor cells (MDSCs) have been shown to be associated with increased tumor growth [11–13]. Recent evidence suggests that endogenous regulatory/suppressor cells invoked in the tumor-bearing state may be largely responsible for preventing effective antitumor immune responses [14]. This paradoxical observation of tumor growth in the face of large numbers of activated, circulating tumor-reactive T cells suggests that their antitumor efficacy is actively attenuated *in vivo*. Evidence for this idea has been accumulating rapidly during the past decade, fueled by the discovery of a number of different immune-regulatory cell types, soluble factors, and mechanisms that limit the strength and duration of anti-tumor immune responses. Nevertheless, it has been well recognized that the detection of immune cells infiltrating tumors, such as memory/effector CD8/CD4 T cells or Treg, correlated with the prognosis in certain types of cancers [7, 9, 15, 16]. A successful cancer vaccine, therefore, needs to enhance tumor-specific protective immune responses despite the presence of suppressor cells. 

 Previously, we have demonstrated that a vaccine platform technology, VacciMax, can be used to therapeutically deliver antigens and effectively eradicate established tumors in a mouse HPV16 and melanoma tumor model [17–19]. More recently, we have developed a new, clinic friendly liposome and oil-based delivery platform called DepoVax (DPX) that is well suited for delivering antigen with limited immunogenicity potential. DPX-0907 is a human DPX-based vaccine, containing 7 HLA-A2-restricted peptides indicated for breast, ovarian, and prostate cancer [20, 21].

 DPX-based vaccines are known to induce strong peptide-specific immune response in preclinical studies in mice and, unlike GM-CSF containing conventional emulsion (CE) vaccines, do not promote proliferation of Treg/Tr1 cells following vaccination [21]. However, the status of tumor-induced immune-suppressor cells in tumor-bearing mice during DPX-vaccine-induced tumor suppression has not been studied previously. The present study examines (i) the levels of immune-regulatory cells in blood and spleen during HPV16-C3 tumor progression and DPX-vaccine-induced tumor suppression and investigates the extent of tumor infiltration by such suppressor cells; (ii) using C3 tumor bearing HLA-A2 transgenic mice with increased frequency of suppressor cells, this study also evaluates the ability of human DPX-0907 vaccine to induce effective cancer peptide-specific immune responses. The results indicate that DPX-based vaccine-induced tumor suppression is also accompanied with reduced suppressor cell accumulation within the tumor tissue and that this vaccine platform can induce potent immune response in tumor-induced immune-suppressive environment. 

## 2. Materials and Methods

### 2.1. Mice and Tumor Model

Female C57BL/6 mice, 6–8 weeks of age, were obtained from Charles River Laboratories (Wilmington, PA). HLA-A*0201/H2D^d^ (AAD) transgenic mice were obtained from Jackson Laboratories (Bar Harbor, ME) and were bred in house. These AAD transgenic mice express a chimeric MHC Class I molecule containing human *α*1 and *α*2 domains from human HLA-A*0201 and the transmembrane/cytoplasmic *α*3 domain from mouse H2D^d^. Mice were housed under filter-top conditions and supplied with water and food *ad libitum*. Institutional animal care guidelines were strictly followed for all experiments. For inducing tumors, mice were implanted s.c. with full-length HPV16 transfected, 0.5 × 10^6^ C3 tumor cell line in the left flank as described earlier [17]. This tumor has been used as a model for epithelial malignances and the mice protected by vaccination are resistant to rechallenge. Tumor growth was monitored every 4-5 days and tumor size was recorded until termination of experiments at 5 to 6 weeks after implantation. 

### 2.2. Peptides, T Helper Epitopes, and Adjuvant

All peptides were synthesized by NeoMPS-Polypeptide (San Diego, CA) at >95% purity. HPV16 E7 (H-2D^b^) peptide RAHYNIVTF_49–57_ (R9F) containing CTL epitope was fused to PADRE containing CD4 T helper epitope to generate the fusion peptide (FP). DPX-based vaccine containing FP peptide and adjuvant was designated as DPX-E7. Human cancer peptide vaccine DPX-0907 containing HLA-A2-restricted, seven breast/ovarian/prostate cancer-associated peptides; their source proteins; peptide locations within each protein; and methods used to identify these CTL epitopes have been described recently by Karkada et al. [21, 22]. For DPX-0907, the modified tetanus toxin peptide A16L (AQYIKANSKFIGITEL_830–844_), with an alanine residue added to its amino terminus to enhance stability [23], was used as a T helper (Th) epitope. Both vaccines were adjuvanted with proprietary polynucleotide-based TLR agonist molecule. 

### 2.3. Vaccine Formulation

 Vaccines were formulated either as a conventional emulsion in Montanide ISA51 or as a proprietary DepoVax formulation. Briefly, for DPX-0907, liposomes were formulated by mixing phosphatidylcholine and cholesterol in a 10 : 1 ratio (w : w) (Lipoid GmBH, Germany) with the seven BO peptides (50 *μ*g each per dose), A16L (25 *μ*g/dose), and a proprietary polynucleotide adjuvant (20 *μ*g/dose) in phosphate buffer (50 mM, pH 7.4). Similarly, DPX-E7 was prepared using FP (5 *μ*g/dose) and the adjuvant (20 *μ*g/dose) during liposome preparation. The liposomal solution was lyophilized and then resuspended in Montanide ISA51 (SEPPIC, France) just before vaccination. Control emulsion (CE) vaccine was prepared by mixing the seven BO peptides, A16L, and 5 *μ*g/dose of GM-CSF, and by emulsifying the mixture with Montanide ISA51 using a 3-way stopcock according to manufacturer's recommendations. Similarly, control CE vaccine for DPX-E7 was prepared with corresponding dose of FP and GM-CSF. 

### 2.4. Vaccination Schedules and Tissue Processing

 Six days following tumor implantations on the left flank and when the tumor is established, mice were immunized s.c. with vaccines in DPX or CE formulations or with PBS as control injections on the right flank near the base of the tail. Injection was given as a single dose in most experiments or as two doses with one week interval for CE formulation. Subsequently, 3 weeks and 5 weeks after implantation, blood, spleen, draining lymph nodes (dLN), and tumor tissues were harvested from mice. For assessing immune response in tumor-bearing AAD mice to peptides in DPX-0907, vaccine-draining lymph nodes were isolated eight days following vaccination. These tumor implanted AAD mice showed comparable tumor size as in PBS injected control C57BL/6 mice at the time DPX-0907 was injected.

### 2.5. Flow Cytometry

Unless stated otherwise, all antibodies for immunofluorescence staining and flow cytometry were obtained from eBioscience (San Diego, CA). Three-color staining was performed for detecting Treg cells; briefly, cells were surface-stained with anti-CD4 and anti-CD25 antibodies followed by intracellular staining for Foxp3 using a staining kit (eBioscience). MDSCs in blood and spleen were detected using two-color staining using antibodies against CD11b and Gr1. IL-10/TGF-*β* secreting Tr1 cells in the spleen were identified by intracellular staining of fixed/permeabilized CD4+/CD8+ T cells using anti-mouse-IL-10 or anti-TGF-*β* antibodies (R&D Systems, Minneapolis, MN), after the cells were stimulated with peptide R9F for 48 h.

 To identify antigen-specific CD8+/CD4+ T cells producing IFN-*γ* following DPX-0907 injection of AAD mice, two-color intracellular staining was performed. dLN cells were cultured overnight in a 24-well plate with peptide-loaded dendritic cells (DCs, see below) at 10 : 1 ratio and 10 *μ*g/mL peptides at 37°C in 5% CO_2_. On the following day, GolgiStop (BD Bioscience) was added to each well and the plates were incubated for additional 4 hours. Surface and intracellular staining of fixed/permeabilized cells was performed as described previously [21]. Stained cells were collected on a FACSCalibur flow cytometer (BD Bioscience) and the data analysis was done using WinList 6.0 software (Verity Software House, Topsham, ME). 

### 2.6. ELISPOT Detection of Immune Response

 Cells were isolated from the femur bone marrow of naïve AAD transgenic mice and cultured in the presence of GM-CSF to generate mature DCs over eight days as described [21]. Mature DCs were loaded with 10 *μ*g/mL of peptide by incubation at 37°C for 18 h. Activated antigen-specific T cells were detected in the draining lymph nodes of mice using standard IFN-*γ* ELISPOT (BD Bioscience) modified as DC-based ELISPOT assay [21]. PMA (5 ng/mL; Sigma) and Ionomycin (1 *μ*g/mL; Sigma) served as positive controls, while stimulation with irrelevant peptide (SVYDFFVWL, T2P-2_180–188_, S9L) and media alone served as negative controls. The plates were developed on the following day for detecting IFN-*γ* secreting cells using standard method and spots were enumerated by three independent personnel or through automated ELISPOT plate reader. 

### 2.7. Tumor MDSCs Enrichment and Their Effect on T Cell Activation

 To study the effect of MDSCs on T cell activation in normal and tumor-bearing mice, tumor infiltrating MDSCs were enriched to >95% purity using MACS column (Miltenyi Biotech GmbH, Germany). Single cell suspension of tumor-derived cells were treated with biotinylated anti-Gr1 antibody, washed, and treated with streptavidin microbeads before sorting on MACS column. Single cell suspensions from LN of normal mice or tumor-dLN of DPX-E7 or PBS injected mice with large tumors were prepared on week 5 after implantation. dLN cells were stimulated using plate bound anti-CD3 antibodies, in the presence of 0.5 *μ*g/mL anti-CD28 antibody, 20 U/mL IL2, and 20 ng/mL IL12. Cell stimulation was carried out for three days in the presence and absence of 1 : 10 ratio of tumor derived MDSCs to dLN cells. Effect of MDSCs on T cell activation was measured by the ability of these cells to generate IFN-*γ* using intracellular cytokine staining of CD8 T cells as described above. 

### 2.8. Cytospins and Fluorescent/Confocal Microscopy

To analyze tumor infiltrating cells, matched volumes of tumor tissues from different groups of mice were homogenized and single cell suspensions were adhered to plastic dishes for 2 hours at 37°C, and 50 *μ*L nonadherent cells were used to prepare cytospin slides. In the initial experiments, single color staining for CD8 or CD25 was performed on cytospin preparations of tumors from DPX-E7, CE, and PBS control groups. To identify functionally relevant, glycoprotein-A-repetitions-predominant- (GARP-) expressing activated Treg cells, slides were double stained with anti-CD25-FITC and anti-GARP-PE antibodies. To visualize regulatory cells within tumors, tumor tissues were snap-frozen in liquid nitrogen, sectioned, acetone-fixed, and stained for Treg and MDSCs using combinations of anti-CD4/anti-CD25 and anti-CD11b/anti-Gr1 antibodies, respectively. Images were captured on a Zeiss LSM 510 Laser Scanning Confocal Microscope. 

### 2.9. Statistical Analyses

 Statistical analyses were performed using a Student's unpaired *t*-test, with *P* values < 0.05 considered significant.

## 3. Results

### 3.1. Tumor Growth and Vaccine-Induced Inhibition

**Figure 1:**
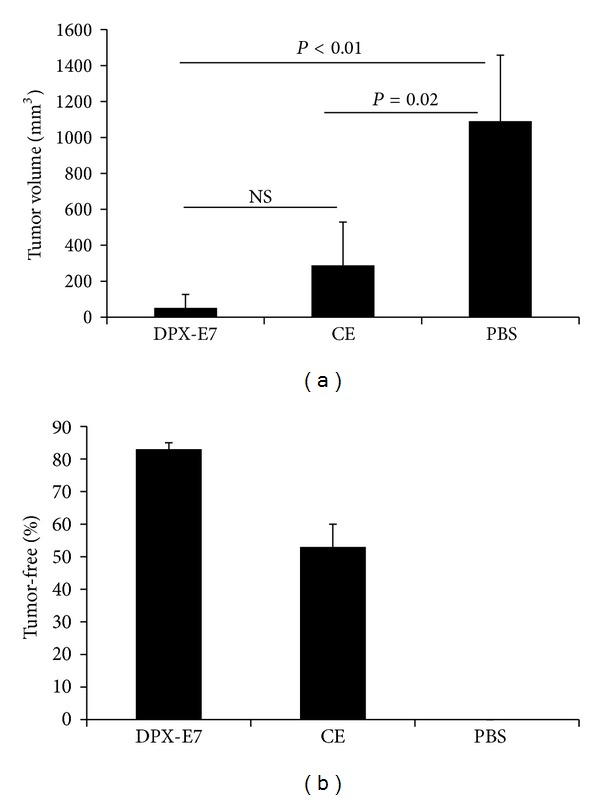
Average tumor volume (a) and percentage of tumor-free C57/BL6 mice (b) at week five after C3 tumor challenge. Mice were either nonvaccinated (PBS control) or vaccinated either with DPX-E7 or CE-based vaccine as outlined in methods, after 6 days of tumor implantation. Tumor measurements were carried out every 3-4 days and tumor volume was calculated. Data represents mean ± SDM from at least 8–10 mice per group and from one of three independent experiments with similar findings.

 Tumor take and tumor growth kinetics for C3 tumors in C57BL/6 mice has been described earlier [17]. AAD transgenic mice, which have the same background, also exhibited similar tumor growth kinetics (data not shown). As shown in Figure 1(a), by week 5 after implantation, PBS control mice developed a mean tumor size of nearly 1000 mm^3^ and CE-immunized mice had the tumors in the range of 200–400 mm^3^ size. In contrast, DPX-E7-immunized mice showed good tumor inhibition with a small percentage of mice developing tumor volume of ≤100 mm^3^. Mean tumor volume was not significantly different between DPX-E7 and CE-vaccinated groups, but control mice had significantly larger tumors compared to both groups of vaccinated mice. Since tumor volume is measured only in surviving mice, to get a better picture on vaccine efficacy we determined tumor free mice in each group. DPX-E7-vaccinated group had most tumor free mice (>80%) while about half the mice in CE vaccine group did develop tumors, and all the mice in PBS control group showed tumor growth (Figure 1(b)). Similar differences between groups of mice in tumor size/tumor-free status were also seen at week 3 after implantation albeit with lesser tumor volume. 

### 3.2. Tumor-Induced Treg Cells and the Effect of Vaccination

**Figure 2:**
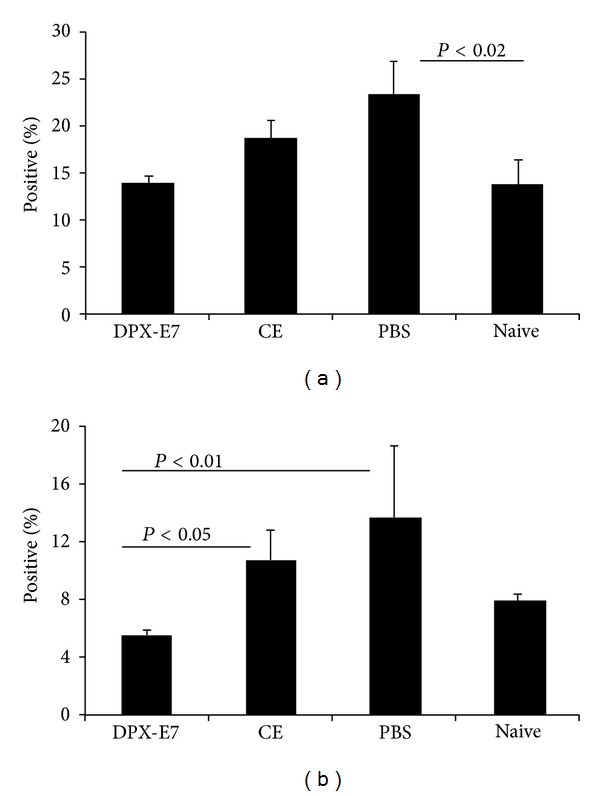
Percentages of Treg cells in spleen (a) and blood (b) of C3 tumor-challenged mice at week five after challenge. Groups of tumor-implanted mice were injected with PBS alone, or vaccinated using DPX-E7 vaccine, or injected as CE vaccine emulsion. Percentages of CD4^+^CD25^+^Foxp3^+^ Treg cells in spleen and blood were determined using flow cytometry. Data represents mean ± SDM from at least 5 mice per group and from one of three experiments with similar findings.

 To investigate tumor-induced changes in Treg cells in blood and spleen, mice were sacrificed at week-3 and week-5 after tumor implantation. Percentage of CD4^+^CD25^+^Foxp3^+^ Treg lymphocytes increased significantly (*P* < 0.02) in non-vaccinated PBS control mice compared to naïve mice (Figure 2(a)). In contrast, level of Treg cells remained significantly lower in DPX-E7 immunized mice over non-vaccinated control mice and was comparable with naïve mice. There was some increase in Treg cells in CE-vaccinated mice but was not significantly different from PBS control or tumor-free naïve mice. However, at three weeks after implantation, level of Treg cells in CE-vaccinated mice was as high as that seen in PBS group and was significantly higher than DPX-E7-vaccinated and naïve mice (*P* < 0.01, data not shown). Similar to the observations in the spleen, circulating Treg cells in the blood also showed increases in PBS control mice and in mice vaccinated using CE formulation (Figure 2(b)). Although mean frequency of Treg was relatively higher in PBS control mice the differences were not statistically significant as compared to naïve mice. However, mice injected with CE vaccine showed significantly higher Treg cells in blood compared to DPX-E7 vaccinated mice (*P* < 0.05) and the level of Treg in the latter group was significantly lower than PBS control mice (*P* < 0.01).

### 3.3. Tumor-Induced MDSCs and the Effect of Vaccination

**Figure 3:**
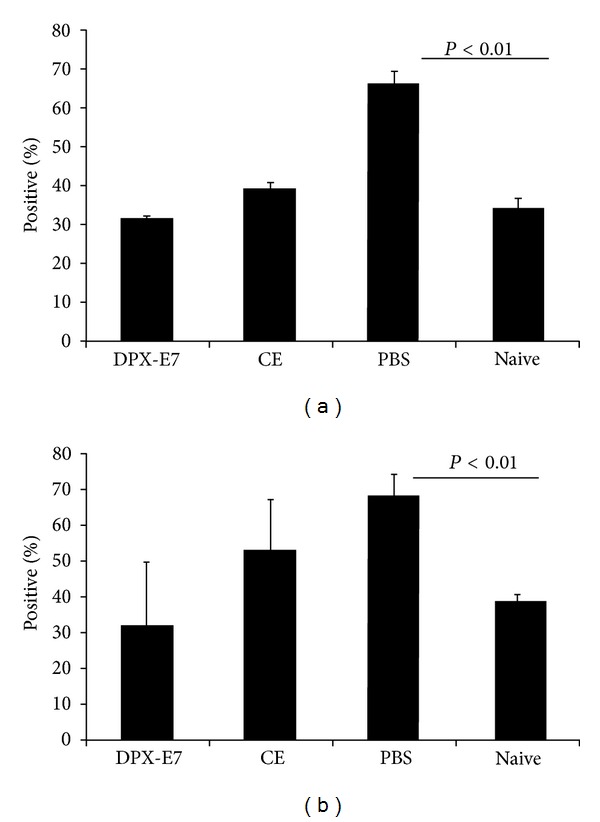
Percentages of MDSCs in the spleen (a) and blood (b) of C3 tumor-challenged mice at week five after challenge. Groups of tumor implanted mice were injected 6 days later with PBS alone, vaccinated using DPX-E7 vaccine, or injected as CE vaccine emulsion. Percentages of CD11b^+^Gr1^+^ MDSCs in spleen and blood were determined using flow-cytometry. Data represents mean ± SDM from 5 mice per group and from one of three experiments with similar findings.

 Similar to Treg cells, CD11b^+^Gr1^+^ MDSCs showed significant increases in PBS injected, tumor-challenged control mice, both in spleen (*P* < 0.01, Figure 3(a)) and blood (*P* < 0.01, Figure 3(b)). Although, conventional vaccine injected mice had some increase in MDSCs levels in the spleen, and more noticeably in the blood, the differences were not statistically significant. In contrast, level of MDSCs in DPX-E7 vaccinated mice remained very close to what was seen in tumor-free naïve mice. Mice that received CE-vaccine also had higher Gr1+ cells in the circulation, but the difference was not significant. 

### 3.4. Effect of Tumor-Induced MDSCs on Effector T Cell Function

**Figure 4:**
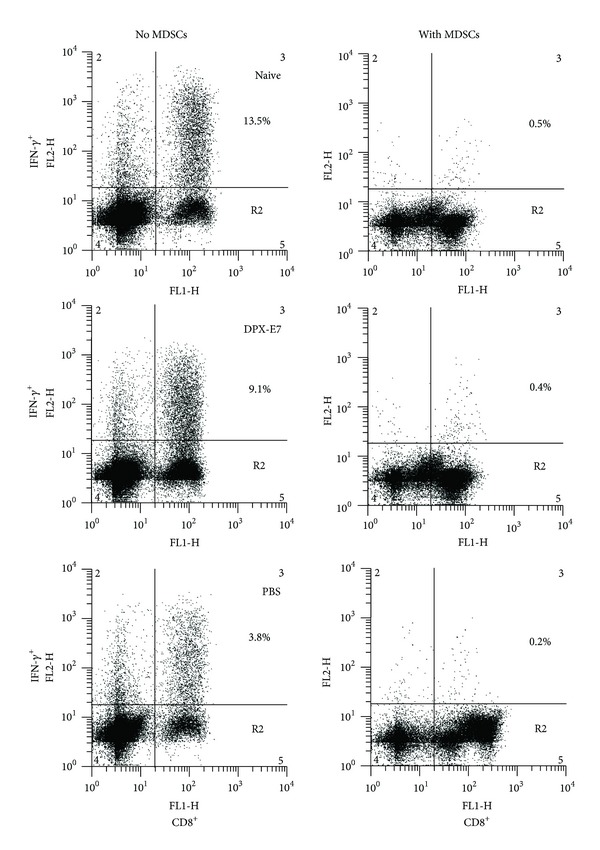
Effect of MDSCs on tumor-dLN T cell activation using anti-CD3/anti-CD28 antibodies. MACS-enriched intratumoral MDSCs were isolated from tumor-bearing mice at week 5 after tumor implantation and were cocultured with tumor-draining lymph node cells from DPX-vaccinated, non-vaccinated controls or LN cells from tumor-free naïve mice during anti-CD3/anti-CD28-mediated T cell activation. Left panel represents T cell activation in the absence of MDSCs and the right panel with MDSCs coculture. IFN-*γ* producing CD8 T cells were detected by intracellular staining as described in methods. Representative histograms are from 4 mice per group, from one of two experiments with similar results are shown.

In order to assess the ability of tumor infiltrating MDSCs to inhibit T cell effector function, enriched MDSCs from large tumors were co-cultured during anti-CD3 activation of tumor-dLN cells. In the absence of MDSCs, CD8 T cell activation was slightly decreased by about 25% in DPX-E7 immunized mice compared to tumor-free naïve mice (Figure 4, left panel; 13.5% versus 9.1%). In contrast, ability of tumor dLN CD8 T cells from non-vaccinated mice to secrete INF-*γ* was markedly compromised (13.5% versus 3.8%). However, in the presence of tumor-derived MDSCs, *in vitro* CD8 T cell activation was almost completely inhibited in all the three groups of mice tested (Figure 4, right panel). 

### 3.5. Antigen-Specific Proliferation of Tr1 Cells

**Figure 5:**
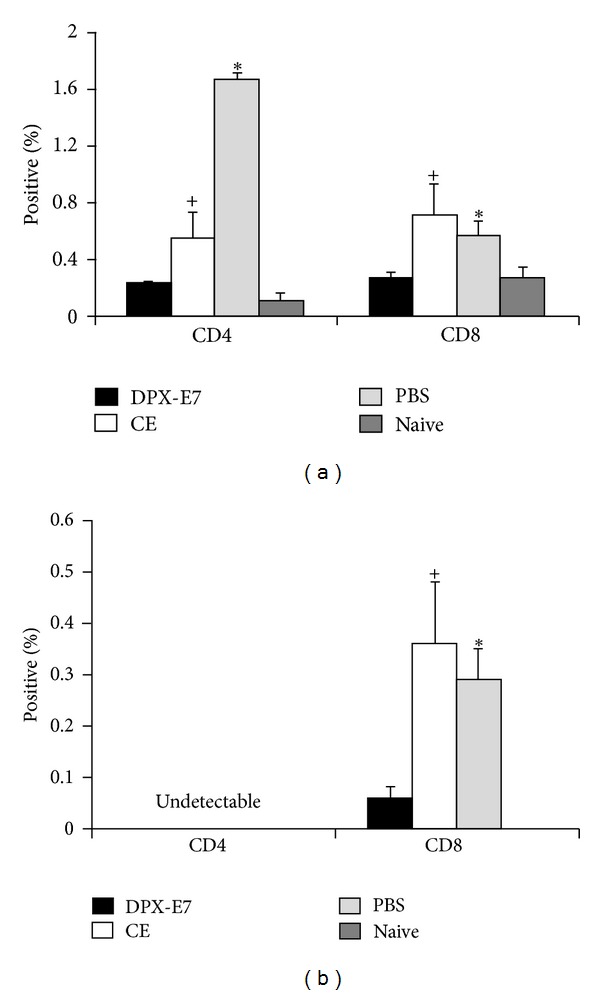
Percentages of spleen CD4 and CD8 T cells stained positive for intracellular IL10 (a) and TGF-*β* (b) from C3 tumor-challenged mice at week five after challenge. Groups of mice were injected with PBS alone, vaccinated against HPV-E7 in DPX platform, or injected as CE vaccine. Spleen cells were stimulated for 72 h with E7 antigenic peptide (R9F, 5 *μ*g/mL) and analyzed for intracellular cytokines using flow-cytometry. Data represents mean ± SDM from at least 5 mice per group and from one of two experiments with similar findings. (a), ^+^ *P* < 0.04, *P < 0.005; (b), ^+^ *P* < 0.05, *P < 0.03.

 We further investigated both CD4 and CD8 T cells from the spleen for their ability to secrete antigen-induced inhibitory cytokines such as IL10 and TGF-*β* in C3 tumor challenge model. As shown in Figure 5(a), at week 5 of tumor growth, frequency of IL10 secreting cells, among both CD4 and CD8 T cells, increased significantly in PBS-treated control mice. Interestingly, vaccination using CE platform also resulted in significantly higher IL10^+^ CD4 and CD8 T cells compared to naïve mice. In contrast, antigen-stimulated spleen cells from DPX-E7 vaccinated mice showed only a marginal and insignificant increase in IL10^+^CD4^+^ cells and did not show any increase in IL10^+^CD8^+^ cells. Unlike IL10 secreting CD4 T cells, we were not able to detect CD4^+^TGF-*β* ^+^ cells in all groups of animals studied (Figure 5(b)). However, a small but significantly higher proportion of CD8 cells in both PBS control mice and CE-vaccinated mice showed intracellular TGF-*β* compared to mice immunized with DPX-E7. No TGF-*β* was detected in CD8 T cells from naïve mice. 

### 3.6. Tumor Infiltrating Treg and MDSCs

**Figure 6:**
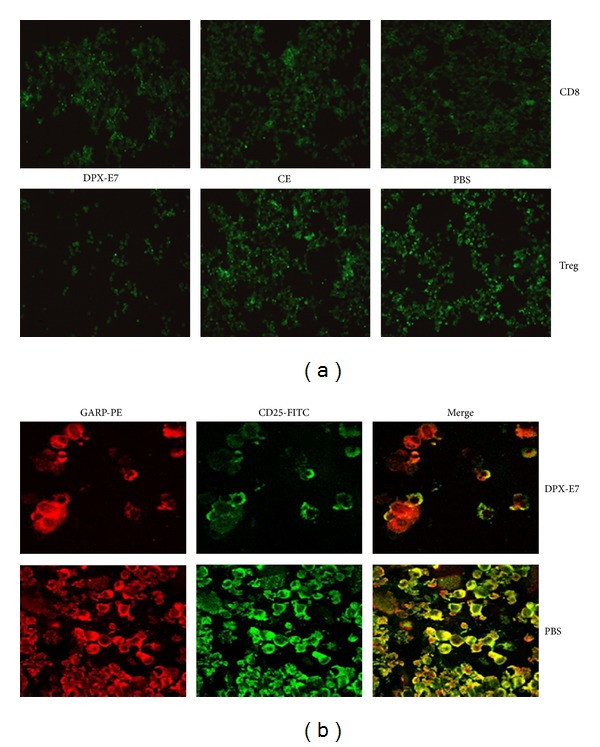
Frequency of intratumoral CD8 T and Treg cells in tumor-challenged mice at week five after challenge. Six days after implantation groups of mice were injected with either PBS alone or vaccinated using DPX-E7 or CE vaccine. Matched volume of tumor tissues was used to prepare single cell suspensions for cytospin slides. In (a), slides were stained with FITC conjugated anti-CD8*β* (upper panel) or anti-CD25 antibodies (lower panel; ×25 magnification). In (b), cytospin smears were double stained with anti-GARP-PE and anti-CD25-FITC antibodies and were analyzed using confocal microscopy to identify double-positive, activated Treg cells (×100 magnification). Smears shown are representative of 5-6 tumors processed in each group and subjected to blinded analysis.

**Figure 7:**
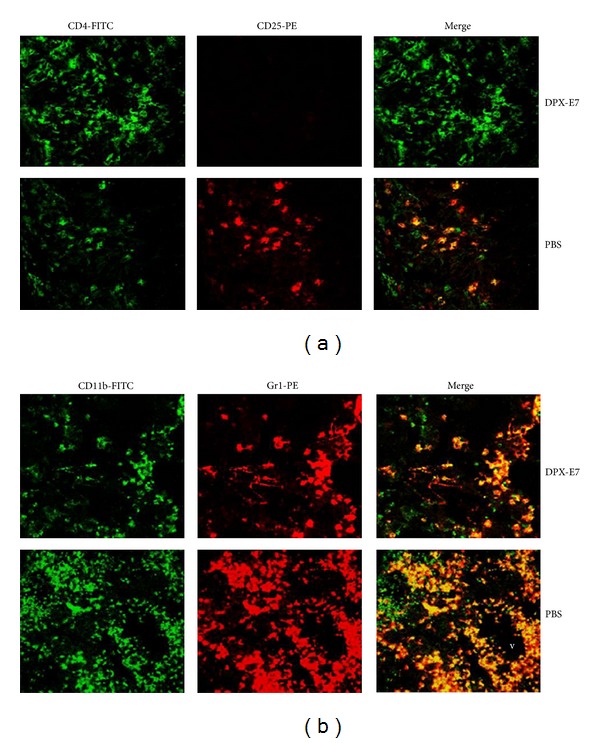
Detection of *in situ* intra-tumoral Treg cells (a) and MDSCs (b) in C3 tumor-challenged mice at week five post implantation. Tumor implanted groups of mice were injected with PBS alone (b), or vaccinated using DPX-E7 (a). Frozen tumor tissues were sectioned, fixed and stained for CD4^+^CD25^+^ Treg and CD11b^+^Gr1^+^ MDSCs, analyzed by confocal microscopy (×40 magnification). v, blood vessel. Representative sections shown are from 5-6 tumors processed and subjected to blinded analysis in each group.

 In order to assess the extent of tumor infiltrating CD8, Treg, and MDSCs, tumor tissues from different groups of mice were analyzed at week 5 of tumor growth. This was done either by isolating cells from tumor tissue for cytospin preparation for immunofluorescence staining (CD8/Treg; Figure 6) or by direct staining of snap-frozen tumor sections (Treg/MDSCs; Figure 7). As shown in Figure 6(a), higher CD8 T cells were found to infiltrate tumors from DPX-E7 immunized mice compared to tumors from CE-vaccinated or PBS control mice (upper panel). In contrast, staining with anti-CD25 antibodies suggested least CD25^+^ T cell infiltration of tumors in DPX-E7 vaccinated group compared to tumors from both CE-vaccinated and control mice (Figure 6(a), lower panel). Further, to identify tumor infiltrating Treg cells as functionally relevant regulatory cells [24], cells were double stained with anti-GARP and anti-CD25 antibodies. Tumors from non-vaccinated PBS control mice had much higher tumor infiltrating CD25^+^GARP^+^ Treg cells compared to DPX-E7-treated tumor-bearing mice (Figure 6(b)).

 Tumor tissues from vaccinated and non-vaccinated mice were also snap-frozen, sectioned, and double stained to identify Treg and MDSCs *in situ*. For Treg cells, since anti-GARP antibody was not compatible to use on frozen/processed/fixed tissues, we had to rely on staining for CD4^+^CD25^+^ T cells. Nevertheless, tumors from DPX-E7 vaccinated mice showed considerably less CD4^+^CD25^+^ double positive cells compared to non-vaccinated mice (Figure 7(a)). Similarly, DPX-E7 treatment also resulted in much reduced CD11b^+^Gr1^+^ MDSCs infiltration of tumors compared to non-vaccinated PBS control mice (Figure 7(b)). Although no attempts were made to positively identify blood vessels in the tissue sections, presenting morphology appears to suggest accumulation of MDSCs in the perivascular tissues, probably migrated out of tumor vasculature (v). 

### 3.7. Efficacy of Human DPX-0907 Cancer Vaccine in Tumor-Bearing AAD Mice

Type 1 CD8^+^ T cell responses are crucial to the ability of the host to prevent the development and progression of cancer [2, 25]. However, it is also critical that vaccines used for immunotherapy need to elicit such CD8 T cell response in cancer-bearing host, most of them with concurrently active immune-suppressive mechanisms. Hence, we assessed the efficacy of DPX-0907 in eliciting IFN-*γ* producing CD8^+^ and CD4^+^ T cells within vaccine dLN 8 days after immunization of late-stage tumor bearing AAD mice. As controls, tumor-free AAD mice were immunized in parallel. At week five after tumor implantation, AAD mice showed similar tumor growth kinetics as C57/BL6 mice (as in Figure 1) with comparable increase in the percentages of spleen Treg (26.8 ± 3.1 versus 14.9 ± 2.4 in naive, *P* < 0.02) and MDSCs (79.1 ± 9.2 versus 43.3 ± 6.4 in naïve, *P* < 0.001). DPX-0907-induced, peptide-specific immune responses were determined by DC-based ELISPOT detection of IFN-*γ* ^+^ cells in dLN and by intracellular staining for INF-*γ* ^+^CD8^+^ and IFN-*γ* ^+^CD4^+^ T cells.

**Figure 8:**
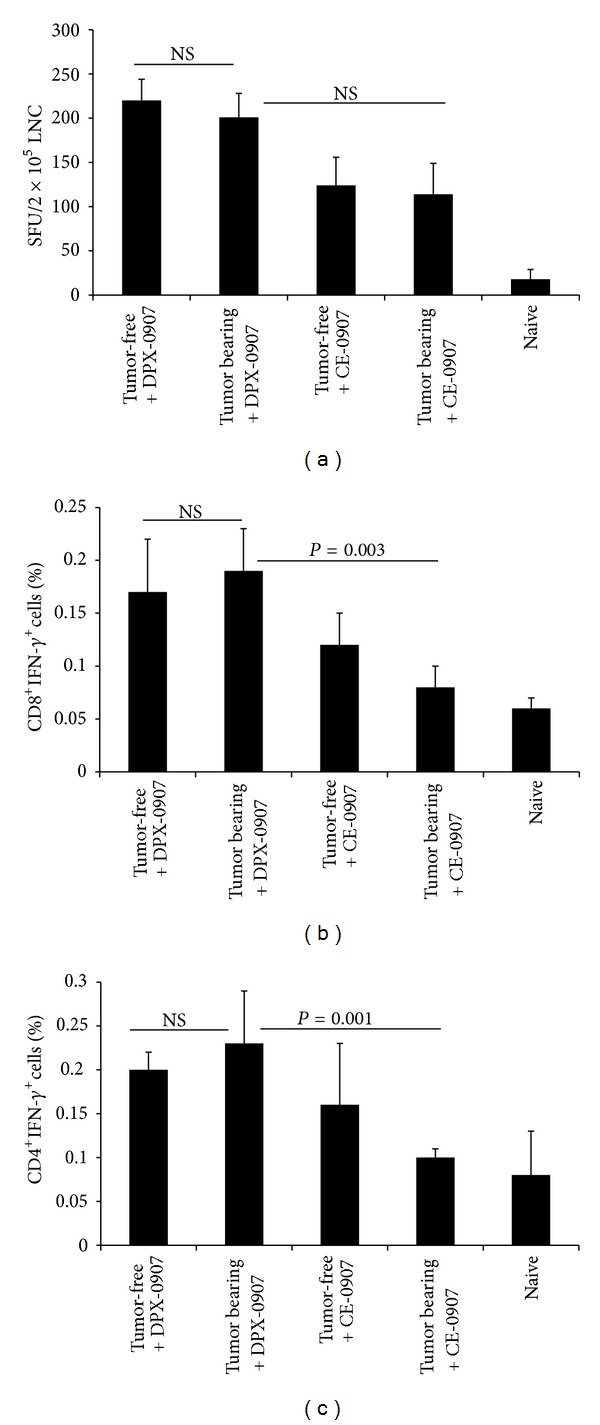
Peptide-specific IFN-*γ* response to DPX-0907 and to a vaccine with peptides in CE platform in tumor-bearing and tumor-free HLA-A2 transgenic mice. Groups of naïve AAD mice were left untreated or vaccinated with DPX-0907 or with the peptides in CE formulation. In parallel, mice bearing week 3 and week 5 C3 tumors were vaccinated with DPX-0907 or CE formulation. Draining lymph node cells were harvested after 8 days after vaccination and analyzed for their ability to secrete IFN-*γ* in response to antigen stimulation in syngenic DC-based ELISPOT assay (a). Data represents mean ± SDM from at least 7 mice per group and from one of two experiments with similar findings. Data from week 3 tumor bearing mice were similar to week-5 tumor-bearing mice (not shown). Intracellular cytokine staining and flow-cytometry, for determining percentages of CD8^+^IFN*γ* ^+^ cells among total CD8 T cells (b) and CD4^+^IFN*γ* ^+^ cells among total CD4 T cells (c) cells, were also performed and the mean percentages ± SDM are shown.

 Following DPX-0907 vaccination, both tumor-bearing and tumor-free AAD mice showed strong and equivalent number of cancer peptide-specific IFN-*γ* secreting cells in the dLN, as seen in ELISPOT assay (Figure 8(a)). In both groups of mice, the peptide-specific immune response was >10-fold higher compared to unstimulated background values. When the peptides are injected in CE formulation, although ELISPOT response was lower than DPX-0907 treated groups, the differences were not statistically significant. Intracellular cytokine staining showed that percentages of INF-*γ* ^+^CD8^+^ T cells from both tumor-free and tumor-bearing, vaccinated mice were much higher than naïve mice (Figure 8(b)), and there was no significant difference in IFN-*γ* ^+^CD8^+^ T cells between tumor free and tumor bearing mice. Intracellular IFN-*γ* response in CD4 T cells was also analyzed, and as shown in Figure 8(c), CD4^+^ cells from DPX-0907-vaccinated tumor-bearing mice and tumor-free mice showed equivalent percentages of IFN-*γ* ^+^ cells. Unlike ELISPOT results, CE-vaccinated group had significantly lower IFN-*γ*-secreting CD8 and CD4 T cells compared to DPX-0907 vaccinated, tumor bearing mice.

## 4. Discussion

 Tumor microenvironment, influenced largely by the growing tumor, is dominated by tumor-induced interactions with host tissues and cells [26]. Although it has been well documented that cancer patients generate antitumor effector T cells, either endogenously as part of anti-cancer immune surveillance or as a result of cancer immunotherapy, tumor inhibition is often hindered by mechanisms that block T cell functions [9, 10, 12–14, 26]. Many cancer researchers believe that the efficacy of immune therapy against established tumors depends on the effector functions of recruited lymphocytes within the tumor bed. Hence, the ability to block tumor escape from immunological targeting depends largely on a better understanding of the cellular and molecular pathways within the tumor microenvironment. In this regard, tumor-induced immune-regulatory/suppressor cells and their recruitment into tumor tissue appear to have a major role in neutralizing antitumor effector mechanisms [10, 13, 14]. The present study examines association of regulatory/suppressor cells during the growth inhibition of HPV16-E7 expressing C3 tumors by a potent vaccine platform, DPX-E7. Results presented here indicate that, in this model, tumor inhibition is also accompanied by decreased Treg/Tr1/MDSCs frequency in the peripheral tissue and reduced infiltration of the tumor by such cells. 

 In addition to Type-1 CD8^+^ T cells playing a crucial role in the efficacy of immunotherapy for cancer patients [25], Th1 CD4^+^ T cells also play a role in regulating antitumor protective efficacy, either by enhancing IFN-*γ*-mediated increase in MHC Class I expression and antigen-presenting capacity of DC and macrophages leading to the formation of effector/memory CD8 T cells or by dampening the immune response via the action of regulatory T cells [14, 27]. However, in many instances, the lack of correlation between the frequency of antigen-specific T cells and prevention of tumor growth suggests that efficacy depends on functional quality of vaccine-activated T cells [5, 28]. In many human cancers such as cervical, ovarian, melanoma, lymphoma, gastrointestinal, and head and neck cancer, level of tumor infiltrating CD8 versus CD4^+^Foxp3^+^ Treg cells provides strong prognostic factor (reviewed in [7, 10]). However, improved prognosis is lost if concurrent accumulation of Treg is also observed in tumor biopsies along with tumor-specific CD8 T cells [9, 15, 29]. In parallel, many studies in mouse models show that depleting Treg cells or reducing their suppressive activity improves spontaneous or immunotherapy-mediated tumor clearance. 

 CD4^+^CD25^+^ natural Treg cells that represent approximately 5% to 10% of peripheral CD4^+^ cells, constitutively express CD25 (IL-2R*α*), glucocorticoid-induced tumor necrosis factor receptor (GITR), CTL antigen 4 (CTLA-4), and the transcription factor forkhead box p3 (Foxp3). Recent phase 3 clinical trial with ipilimumab blockade of CTLA-4 has shown some clinical benefits in stage III/IV melanoma patients, suggesting the potential of suppressor cell inhibition in promoting endogenous anti-tumor immune responses [30]. The exact mechanism of action of Treg cells is still under debate, as is the nature of their antigen recognition. However, it should be noted that tumor antigen-specific Treg cells occur naturally in certain metastatic cancers [31, 32] and can also be induced by vaccination [33]. 

As shown in this study, tumor infiltrating Treg cells were at their lowest level in DPX-E7 vaccinated group, which also had much lower tumor burden. Two possible explanations can be put forward for enhanced efficacy of DPX-based vaccine platform. Firstly, if a proportion of suppressor cells are induced by growing tumor itself, their number is reduced in DPX-E7-treated mice over CE-treated mice because of effective control of tumor volume in the former group. Secondly, it is possible that a proportion of regulatory cells are antigen driven, as has been shown in non-vaccinated and HPV16 peptide vaccinated cervical cancer patients [34, 35]. DPX-based vaccines use lyophilized liposomes containing the antigen (s) and adjuvant, which are used as carriers that allow for the incorporation of hydrophilic antigens and adjuvant directly into an oil medium. Moreover, these vaccines form a depot of antigen/adjuvant at the site of injection and gradually release antigens and adjuvant to be taken up by antigen-presenting cells that are shown to accumulate around the vaccine injection site [21]. It is plausible that, unlike CE vaccinated mice, gradual antigen uptake and processing in DPX-E7 group would allow strong Type 1 response without enhancing antigen-driven regulatory cell responses. Enhanced suppressor/regulatory cell expansion could be associated with high antigen exposure over short periods of time. Furthermore, use of GM-CSF as adjuvant in CE formulation, to mimic the most common approach in designing human cancer vaccine delivery system in recent years, may also lead to increased Treg/MDSCs proliferation. It is interesting to note that the immunosuppressive strength among MDSCs subsets, during CD8 T cell activation, is determined by GM-CSF [36]. Recently, we made similar observations using human cancer peptide immunization of AAD mice using CE formulation [21].

 The regulatory and immune-suppressor cells associated with cancers are diverse, and quite often mere expression of certain phenotypic markers does not necessarily parallel their inhibitory functions. In this regard, the present study attempted to recognize functionally relevant regulatory and suppressor cells by using anti-GARP antibodies to recognize activated Treg cells. It has been well documented that expression of GARP is necessary for their suppressive function, possibly related to its role as cell surface receptor for TGF-*β* and its ability to induce Foxp3 expression [24, 37]. 

MDSCs recruitment to tumor tissues most likely results in the inhibition of effector CD8 T cells within tumor microenvironment. Although we have not tested the inhibitory function of MDSCs derived from a noninflammatory tissue, the finding of strong suppressive function of/inflammatory site-derived MDSCs is in agreement with a recent report [38]. We are currently investigating the status of MDSCs/Treg in breast, ovarian, and prostate cancer patients receiving DPX-0907 and survivin-targeted DPX-Survivac peptide cancer vaccine in ovarian cancer patients. It may be feasible to correlate clinical benefits of such DPX-based cancer vaccines and their influence on the frequency of suppressor cells in these patients. Similar studies with simultaneous determination of immune response and suppressor cell analysis would validate the ability of DPX-based vaccines to specifically induce potent anti-tumor immune response.

 In preclinical studies DPX-0907 has shown potent peptide-specific immune response in HLA-A2 transgenic mice [21]. However, its ability to induce comparable immune response in tumor-bearing mice with expanded suppressor cell populations has not been investigated previously. This aspect is important since many cancer patients with minimal residual disease, even after chemotherapy and radiation, are expected to be in immune-suppressed state. An effective therapeutic vaccine is expected to induce effective immune response under such adverse conditions. Current study using tumor-bearing HLA-A2 transgenic mice shows that DPX-0907, a potent adjuvented vaccine platform, is able to induce antigen-specific immune response which is not compromised in the presence of tumor-induced immune-suppressor cells. Although C3 tumor model is unrelated with respect to DPX-0907 included antigens, the nonspecific nature of immune suppression would have played an inhibitory role, had DPX-0907 been not a potent vaccine platform. Nevertheless, inclusion of other drugs aimed at reducing suppressor cell frequency, in combination of DPX-based vaccines, might alleviate the negative effect of such cells within the tumor bed. Efforts are currently underway to assess DPX-0907 in a mouse tumor model that expresses both HLA-A2 and DPX-0907 included antigen(s). In a therapeutic setting, these findings are expected to provide insight into the effectiveness of DPX-based vaccines in immune-suppressed cancer patients.

## 5. Conclusions

The present study provides additional information on tumor-induced suppressor cells and the advantages of using DPX-based vaccines over CE-based vaccines as a means of immunotherapy. Results from on-going clinical studies with survivin-targeted cancer vaccine DPX-Survivac and from animal studies in relevant tumor-bearing HLA-A2 transgenic mice are expected to contribute to our understanding of the mechanisms involved in enhanced protection provided by this vaccine platform. Full comprehension on the role of Treg/MDSCs and other suppressor cells might facilitate the development of successful intervention strategies for the immunotherapy of cancer with long-term efficacy.
